# Supplementary material for: Splice-Junction-Based Mapping of Alternative Isoforms in the Human Proteome
Source: Cell Rep. Author manuscript; Available in PMC 2020 Jan 15. (PMC6961840; doi:10.1016/j.celrep.2019.11.026)
Supplement: 2 [file NIHMS1546469-supplement-2.zip › DF1/PXD010154/Lung/Lung_10_USP54_TCYDSEDSGHLTDSECNQK.pdf]

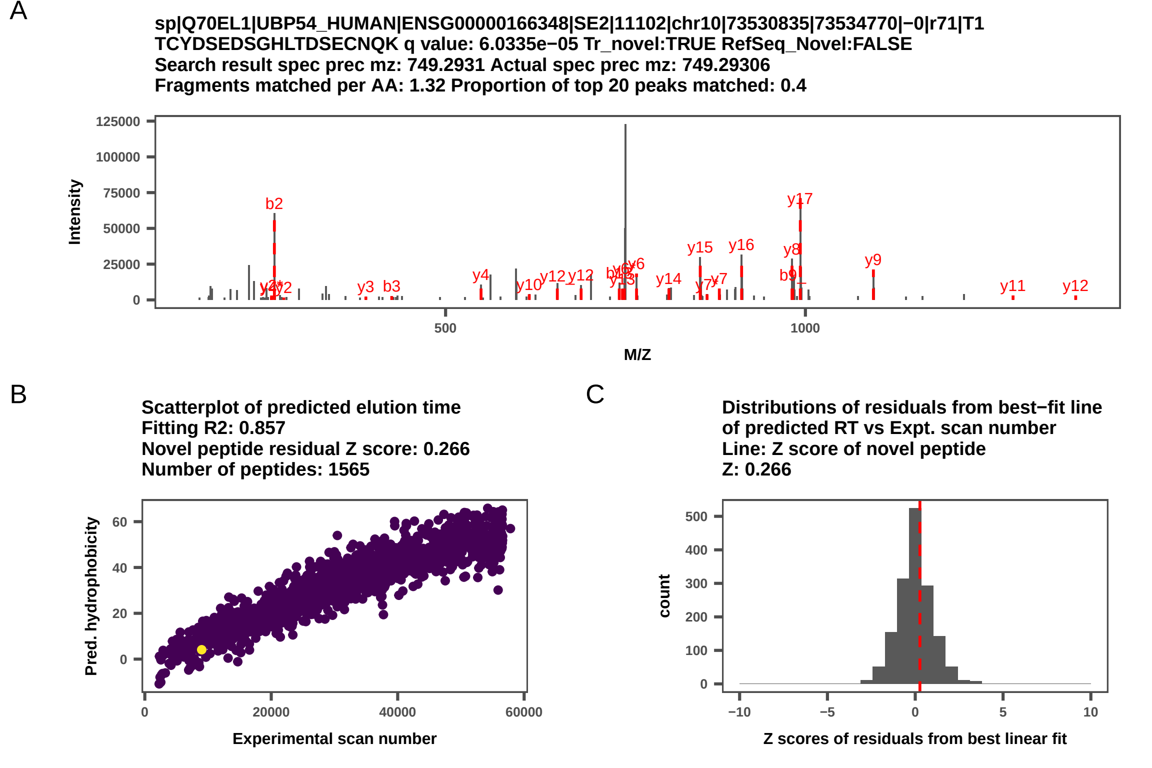
A
sp|Q70EL1|UBP54_HUMAN|ENSG00000166348|SE2|11102|chr10|73530835|73534770|−0|r71|T1
TCYDSEDSGHLTDSECNQK q value: 6.0335e−05 Tr_novel:TRUE RefSeq_Novel:FALSE
Search result spec prec mz: 749.2931 Actual spec prec mz: 749.29306
Fragments matched per AA: 1.32 Proportion of top 20 peaks matched: 0.4
Intensity
125000
100000
75000
50000
25000
0
b2
y3
b3
y4
y10
y12
y14
y15
y16
y17
y8
b9
y9
y11
y12
500
1000
M/Z
B
Scatterplot of predicted elution time
Fitting R2: 0.857
Novel peptide residual Z score: 0.266
Number of peptides: 1565
Pred. hydrophobicity
60
40
20
0
0
20000
40000
60000
Experimental scan number
C
Distributions of residuals from best−fit line
of predicted RT vs Expt. scan number
Line: Z score of novel peptide
Z: 0.266
count
500
400
300
200
100
0
−10
−5
0
5
10
Z scores of residuals from best linear fit
